# Surfactin inhibits enterococcal biofilm formation via interference with pilus and exopolysaccharide biosynthesis

**DOI:** 10.1186/s12866-025-03786-y

**Published:** 2025-02-24

**Authors:** Chun-Yi Wu, Hung-Tse Huang, Yu-Ting Chiang, Kung-Ta Lee

**Affiliations:** 1 Department of Biochemical Science and Technology, National Taiwan University, Taipei, Taiwan; 2 National Research Institute of Chinese Medicine, Taipei, Taiwan

**Keywords:** Surfactin, *Enterococcus faecalis*, Biofilm inhibition, *Bacillus subtilis* subsp. Natto

## Abstract

*Enterococcus faecalis* is a significant pathogen in healthcare settings and is frequently resistant to multiple antibiotics. This resistance is compounded by its ability to form biofilms, dense bacterial communities that are challenging to eliminate via standard antibiotic therapies. As such, targeting biofilm formation is considered a viable strategy for addressing these infections. This study assessed the effectiveness of surfactin, a cyclic lipopeptide biosurfactant synthesized by *Bacillus subtilis* natto NTU-18, in preventing biofilm formation by *E. faecalis*. Analytical characterization of surfactin was performed via liquid chromatography‒mass spectrometry (LC‒MS). Additionally, transcriptomic sequencing and quantitative PCR (qPCR) were used to investigate alterations in *E. faecalis* gene expression following treatment with surfactin. The data revealed notable suppression of crucial virulence-related genes responsible for pilus construction and exopolysaccharide synthesis, both of which are vital for *E. faecalis* adhesion and biofilm structure. Functional tests confirmed that surfactin treatment substantially reduced *E. faecalis* attachment to Caco-2 cell monolayers and curtailed exopolysaccharide production. Moreover, confocal laser scanning microscopy revealed significant thinning of the biofilms. These observations support the potential utility of surfactin as a therapeutic agent to manage biofilm-associated infections caused by *E. faecalis*.

**Supplementary Information:**

The online version contains supplementary material available at 10.1186/s12866-025-03786-y.

## Introduction

*Enterococcus faecalis* (*E. faecalis*) is a facultatively anaerobic, gram-positive bacterium that is commonly found in the gastrointestinal tracts of humans and animals. *E. faecalis* is a significant causative agent of hospital-acquired infections. It predominantly leads to conditions such as urinary tract infections, bloodstream infections, prosthetic joint infections, intra-abdominal pelvic infections, and endocarditis [1, 2]. *E. faecalis* strains have developed resistance to a wide range of clinical antibiotics, including macrolides, tetracyclines, aminoglycosides, and glycopeptides. Crucially, these strains also exhibit resistance to vancomycin, which has traditionally been used as a last-resort antibiotic for treating enterococcal infections. This resistance significantly complicates the therapeutic management of such infections [3]. The emergence of vancomycin resistance makes treating *E. faecalis* infection a significant challenge for clinicians.

The main mechanism by which *E. faecalis* resists to antibiotics is its ability to form biofilms. These biofilms, which are complex structures that adhere to surfaces, significantly increase the resistance of bacteria to antimicrobial agents. Biofilms are resistant to desiccation, extreme pH, and extreme temperatures [4]. Compared with those produced by planktonic cells, biofilms produced by *E. faecalis* exhibit a remarkable increase in antibiotic resistance, ranging from 10 to 1000-fold [5, 6]. Additionally, the formation of biofilms by *E. faecalis* is linked to the induction of persistent inflammation, further complicating the treatment and management of infections caused by this bacterium. This dual challenge of heightened antibiotic resistance and a propensity to cause sustained inflammatory responses underscores the need for innovative therapeutic strategies to target *E. faecalis* biofilms. Biofilms formed by enterococci significantly diminish the efficacy of existing antibiotic therapies. Moreover, these enterococcal biofilms serve as reservoirs for the transmission of bacteria and the dissemination of genes that confer antibiotic resistance [7]. Furthermore, biofilms provide an optimal environment for *E. faecalis* to facilitate horizontal gene transfer of antibiotic resistance genes [8, 9]. Consequently, the development of novel antibiofilm agents targeting *E. faecalis* biofilm formation is of paramount importance.

Produced by *Bacillus* species, surfactin is a potent biosurfactant and an amphiphilic lipopeptide recognized for its extensive biological activities. Its molecular structure is characterized by a cyclic heptapeptide headgroup consisting of the sequence Glu-Leu-D-Leu-Val-Asp-D-Leu-Leu, linked to a β-hydroxy fatty acid chain that varies in length from C12 to C15, via a lactone bond [10]. This compound has garnered increasing attention because of its array of bioactive properties, including antibacterial [11], antifungal [12, 13], antiviral [14, 15], and anticancer [16] properties, as well as its ability to improve type 2 diabetes and insulin resistance [17, 18]. Surfactin targets cell membranes, leading to physical disruption [11], which makes it challenging for microorganisms to develop resistance against it [19, 20]. Previous research has indicated that its oral median lethal dose (LD50) in mice exceeds 5000 mg/kg, suggesting low-to-negligible acute toxicity [21]. These attributes render surfactin a promising candidate for addressing various global public health challenges.

In this study, surfactin was purified, its identity was verified, and its effects on the formation of *E. faecalis* biofilms were investigated via a safranin biofilm assay, adhesion analysis, and 3D morphological analyses. Additionally, variations in exopolysaccharide production were analyzed to understand changes in biofilm structure. Furthermore, the potential molecular mechanisms underlying its inhibitory effects were explored via transcriptomic sequencing (RNA-seq).

## Materials and methods

### Bacterial strains, media, and surfactin production conditions

The *E. faecalis* strains OG1RF and OG1RF::p23cfp used in this study were obtained from the laboratory of Professor Gary M. Dunny (University of Minnesota, USA) [22]. *B. subtilis* natto NTU-18 (BCRC 80390), which was isolated from a commercial natto product, has been preserved and maintained in our laboratory [23, 24]. *E. faecalis* and *B. subtilis* natto were cultured at 37 °C in TSB media (tryptone 17 g/L, soytone 3 g/L, NaCl 5 g/L, K_2_HPO_4_ 2.5 g/L, and glucose 10 g/L), with the pH adjusted to 7.4, under both static and shaking conditions.

For the production of surfactin, the culture conditions were optimized by supplementing YG media (yeast extract, 20 g/L; glucose, 40 g/L) with 2 mM FeSO_4_ to increase the surfactin yield [25]. The culture was incubated at 37 °C for 72 h with shaking at 120 rpm.

### Extraction and purification of surfactin

The surfactin compounds produced by NTU-18 were obtained from the culture supernatant through a combination of acid and solvent extraction, followed by purification via macroporous adsorption resins (MARs). Briefly, after culturing in TSB-FeSO_4_ for 48 h, the culture medium was centrifuged at 8000 × g for 15 min at 4 °C to remove microbial cells. Subsequently, 6 N hydrochloric acid was added to the supernatant to acidify it to a pH of 2. The acidified supernatant was incubated overnight at 4 °C to precipitate the crude surfactin [26]. The pellet was washed with Milli-Q water twice to remove hydrogen ions [27]. The precipitate was then lyophilized, extracted with 100% methanol, centrifuged at 10,000 × g, and subsequently lyophilized again. Diaion HP-20 resins were purchased from Mitsubishi Chemical Group and treated according to the manufacturer’s instructions. Prior to use, the resin was soaked in methanol, washed with Milli-Q water, and dried at 40 °C. A total of 150 g dried resin was packed into a chromatographic column. Subsequently, 2% (v/v) crude surfactin dissolved in methanol was loaded onto the column. The column was washed with 300 mL Milli-Q water, followed by 20% methanol [28], to remove impurities. Surfactin was ultimately eluted with 100% methanol and then lyophilized to obtain surfactin samples. The final purity of surfactin was approximately 70.1% after purification.

### HPLC and HPLC-MS conditions

The surfactin standard (Sigma‒Aldrich, St. Louis, USA) and surfactin compounds produced by NTU-18 were examined via high-performance liquid chromatography with ultraviolet detection (HPLC-UV) and liquid chromatography quadrupole time-of-flight mass spectrometry (LC-Q-TOF-MS) analyses, as described in previous studies [29, 30]. Briefly, the analyses were conducted via a quadrupole time‒of‒flight liquid chromatography mass spectrometer (LCMS-9030, Shimadzu, Kyoto, Japan) equipped with an electrospray ionization (ESI) source. Surfactin was detected in positive-ion mode in the range of 850–1200 m/z. The analysis parameters in positive ion mode were as follows: nebulizing gas - nitrogen; nebulizing gas flow − 2.0 L/min; drying gas flow − 10 L/min; heating gas flow − 10 L/min; interface temperature − 350 °C; and desolvation line temperature − 400 °C. The obtained data were analyzed via LabSolutions software (Shimadzu, Kyoto, Japan). Surfactin separation was achieved via a Shimadzu Shim-pack Scepter C18-120 column (1.9 μm, 2.1 × 100 mm). The separation process utilized a mobile phase consisting of solvent A (0.1% formic acid) and solvent B (acetonitrile) with isocratic gradient elution. A mixture of 30% A and 70% B was used for 20 min at a flow rate of 0.3 mL/min.

### Antimicrobial and antibiofilm activity of surfactin

To establish the minimum concentration required to inhibit bacterial growth, the minimum inhibitory concentrations (MICs) of surfactin against *E. faecalis* were determined via a methodology adapted from previous studies [31] with some modifications, using concentrations ranging from 3.9 µg/mL to 1000 µg/mL. Briefly, cultures of *E. faecalis* at 1 × 10^8^ CFU/mL were exposed to various concentrations of surfactin. The MICs, identified 24 h postincubation, were determined through visual inspection; specifically, the lowest concentrations at which no visible growth occurred were noted. A bacterial biofilm formation assay was performed on a 96-well polystyrene plate as described in a previous study, with some modifications [9]. Briefly, overnight cultures of *E. faecalis* were seeded at 0.1% confluence in TSB medium supplemented with either 10% surfactin or PBS. One hundred microliters of the diluted culture were dispensed into each of five wells for each treatment. The plates were then incubated at 37 °C for 24 h in a SpectraMax^®^ 190 microplate reader (Molecular Devices, LLC, USA), with optical density measurements taken every 2 h at 600 nm to monitor cell growth. After 24 h of incubation, the culture medium was discarded, and the wells were washed twice with double-distilled water (ddH_2_O) and left to air dry for 2.5 h. The biofilm biomass was subsequently stained with a 0.1% safranin solution for 20 min, followed by five washes with ddH_2_O and air drying. The safranin-stained biofilms were quantified by measuring the optical density at 450 nm. The biofilm index was assessed as the ratio of the safranin-stained biomass to cell growth. To comparatively evaluate biofilm formation, the relative biofilm biomass values were normalized to those of the negative control group, which did not receive surfactin.

### Cell culture

The human colon adenocarcinoma-derived Caco-2 cell line was obtained from the Bioresource Collection and Research Center (BCRC, Taiwan). These cells were maintained in Dulbecco’s modified Eagle’s medium (DMEM containing 4.5 g/L glucose) (Gibco, USA) supplemented with 10% FBS (Gibco, USA) and incubated at 37 °C in an atmosphere of 95% humidity and 5% CO_2_. To assess bacterial adhesion, an in vitro assay was conducted on Caco-2 cells using a modified version of that used in a previous study [32]. To establish Caco-2 cell monolayers for the assay, 0.5 mL of cell suspension containing 4 × 10^5^ cells/mL was seeded into each of five duplicate wells in a 24-well plate. The plate was incubated in a cell culture incubator until the cells reached full confluence. Next, the cells were washed with PBS, and the culture medium was replaced with 0.45 mL of DMEM supplemented with 10% FBS. To each well, 0.05 mL of either surfactin or PBS was added. Overnight cultures of *E. faecalis* were then centrifuged, washed with PBS containing 2 mM EDTA, and resuspended in DMEM supplemented with 10% FBS. An aliquot of the *E. faecalis* culture, containing approximately 10^6^ cells, was used to inoculate the Caco-2 cell monolayers. To determine the total bacterial count in the inoculum, the same count of *E. faecalis* culture was added to a control medium consisting of 90% DMEM, 10% FBS, and 10% PBS without Caco-2 cells. The coculture of *E. faecalis* and Caco-2 cells was then incubated at 37 °C in an atmosphere containing 5% CO_2_ for 3 h. Following incubation, the culture medium was discarded, and the infected Caco-2 cells were washed three times with PBS. The cells were then lysed with 0.1% Triton X-100. Serial dilutions of the lysates were plated on selective Todd Hewitt broth (THB) agar plates (Neogen Corp., USA) supplemented with 50 µg/mL rifampicin to quantify the adherent *E. faecalis* cells.

### Experimental setup for scanning electron microscopy (SEM)

*E. faecalis* overnight cultures were diluted at a 1:100 ratio and placed into a 24-well plate, with each well containing 1 mL of medium and a sterile cover glass. The plate was then incubated at 37 °C for 24 h. After incubation, the biomass attached to the cover glasses was subjected to a two-step fixation process. Initially, it was prefixed with a mixture of 2.5% glutaraldehyde and 2.5% paraformaldehyde in 0.05 M cacodylate buffer at 25 °C for 1 h. This was followed by a triple rinse with 0.05 M cacodylate buffer. The samples were then postfixed with 1% osmium tetroxide in the same buffer at 25 °C for another h. After fixation, the samples underwent chemical dehydration through a graded series of ethanol concentrations (30%, 50%, 70%, 85%, 90%, 95%, and 100% [twice]). The samples were subsequently prepared for microscopic analysis via a CO_2_-based critical point dryer and ion coater. The final examination of these dehydrated samples was conducted via an FEI Inspect S SEM operating at a high voltage of 15 kV. The samples were observed at magnifications of 1000×.

### Experimental setup for confocal microscopy

Overnight cultures of *E. faecalis* OG1RF::p23cfp were inoculated at a 1% concentration in TSB with or without surfactin. These cultures were then placed on glass coverslips in 35 × 12 mm tissue culture dishes (Alpha Plus Scientific Co., Taiwan). The dishes were incubated at 37 °C for 24 h. Following the incubation period, the culture medium was discarded, and the biomass adhering to the glass coverslips was washed twice with PBS to eliminate nonadherent cells. The biomass was subsequently fixed with 2% paraformaldehyde (PFA) for 10 min at 4 °C. Following fixation, the biomass adhering to the glass coverslips at the bottom of each dish was imaged via a Leica TCS SP8 X white light laser confocal microscope (Leica Microsystems, Ltd., Germany). Analysis of the images was conducted via Leica Application Suite X (LAS X) software.

### Extraction of exopolysaccharides from the cell suspension and analysis

The extraction and analysis of *E. faecalis* exopolysaccharides were performed via a modified version of the established method [33]. Overnight cultures of *E. faecalis* were inoculated with 1% surfactin in TSB broth, with or without the addition of surfactin. Ten milliliters of the diluted *E. faecalis* culture was then transferred to a tube and incubated at 37 °C. After 24 h of incubation, the culture was centrifuged at 5000 × g for 20 min at 4 °C, and the biomass was washed twice with PBS. The biomass was then resuspended in 2 ml of an aqueous solution containing 0.85% NaCl and 0.22% formaldehyde and incubated at 80 °C for 30 min to extract exopolysaccharides. These exopolysaccharides, which were solubilized in the formaldehyde solution, were subsequently recovered by further centrifugation at 15,000 × g for 30 min at 4 °C. The concentration of polysaccharides was quantified via the phenol‒sulfuric acid (PSA) method [34].

### RNA purification and sequencing

An overnight culture of *E. faecalis* OG1RF was inoculated at 1% in TSB and incubated at 37 °C for 4 h. This was followed by either the addition of surfactin or no treatment, with further incubation at 37 °C for 1 h. After incubation, 0.6 mL of the bacterial culture was combined with 1.2 mL of RNAprotect Bacteria Reagent (Qiagen Ltd., Germany) and incubated at room temperature for 10 min. The cells were then centrifuged at 4 °C for 10 min and stored at -80 °C until RNA extraction. For RNA extraction, the cells were lysed with 30 mg/mL lysozyme and 500 U/mL mutanolysin in Tris-EDTA (TE) buffer at 37 °C for 10 min [35, 36]. Total RNA was subsequently extracted via an RNeasy Mini Kit (Qiagen Ltd., Germany) according to the manufacturer’s instructions. The RNA quality and concentration were measured via an ND-1000 spectrophotometer (NanoDrop Technologies, Inc., USA), and its integrity was verified via a Bioanalyzer 2100 (Agilent Technologies, Inc., USA). Ribosomal RNA (rRNA) was depleted via an Illumina Ribo-Zero Plus rRNA Depletion Kit (Illumina Co., USA). The cDNA library was then prepared from the rRNA-depleted mRNA via a SureSelect Strand Specific RNA Library Preparation Kit (Agilent Technologies, Inc., USA) according to the manufacturer’s guidelines. RNA sequencing was performed on an Illumina NovaSeq X Plus platform (paired-end) by Genomics Co. Ltd., Taiwan.

### RT-qPCR

cDNA was synthesized as described previously. Approximately 500 ng total RNA was used for cDNA synthesis via an Easy Fast RT kit (BIOTOOLS Co., Ltd., Taiwan). The synthesized cDNA was diluted 1:40 with ddH_2_O, and 1 µL of this dilution was used for RT‒qPCR. RT‒qPCR was performed using SYBR^®^ Green Supermix (Hong Da Life Science Co. Ltd., Taiwan) on a Bio-Rad CFX96 instrument (Bio-Rad Laboratories, Inc., USA). Each well contained a total reaction volume of 15 µL, which included 1 µL of a gene-specific primer mixture at a concentration of 10 µM. Reactions were conducted in triplicate to obtain cycle threshold (CT) values. Relative quantification of gene expression was performed via the 2^−ΔΔCT^ method [37]. Gene expression levels were normalized against those of the housekeeping gene *gyrB* [38]. The sequences of the primers used are detailed in Table 1.

**Table 1:** To demonstrate reproducibility, five biological replicates were conducted

Primer target (name)		Sequence (5’-3’)	Reference
Locus tag	Symbol		
OG1RF_RS00035	*gyrB*	Forward 5’-CCTATCGGCCTCGGCTTAG-3’	(Chatterjee et al., 2013) [38]
		Reverse 5’- AGCGAAAGACAGGTGAGAATCC-3’	
OG1RF_RS03530	*bopD*	Forward 5’-AGACGGACACCATTCGTCAA-3’	This study
		Reverse 5’-ACAAGCATTGACATCGACACG-3’	
OG1RF_RS04545	*ebpR*	Forward 5’-TGGTCGTTGACGTTTTTGCC-3’	This study
		Reverse 5’-ATTAAACAGCGTTGGGGCGA-3’	
OG1RF_RS04550	*ebpA*	Forward 5’-GCCAGCTACTTTGAGAGCGA-3’	This study
		Reverse 5’-GGCACCACCATCTTTATTCCC-3’	
OG1RF_RS04555	*ebpB*	Forward 5’-TTAGCAGAAACCGGTGCAATG-3’	This study
		Reverse 5’-TCTCGTTGCTGCGAATCTTTG-3’	
OG1RF_RS04560	*ebpC*	Forward 5’-GTCGTCCGTGATCAAAACAGC-3’	This study
		Reverse 5’-CCAAGTTGCTGCTTTCGTTGT-3’	
OG1RF_RS04565	*srtC*	Forward 5’-CATTACTTTTAATTGCCTGTGCGT-3’	This study
		Reverse 5’-ACTACTTTGGTTTTCTGGTCGT-3’	
OG1RF_RS04580	*entV*	Forward 5’-AGCTGCACAAAAGAAAGCCTG-3’	This study
		Reverse 5’-TAGCCCACATTGAACTGCCC-3’	
OG1RF_RS07835	*gelE*	Forward 5’-CTTGGTTGGTTTACCTGAATGTCT-3’	This study
		Reverse 5’-CAGTGGTGTCAGCAGCCTTT-3’	
OG1RF_RS08730	*glnA*	Forward 5’-GTGAACGATTTCTGCCGCTC-3’	This study
		Reverse 5’- ACATGCCCGTGCCTATACTG-3’	
OG1RF_RS08790	*epaX*	Forward 5’-AGCCACCGCATAAACAGATG-3’	This study
		Reverse 5’-TTCTGGTCAAGAGCCTGAGA-3’	
OG1RF_RS08795	*epaOX*	Forward 5’-GCCACTGTCATCAGAAGAACC-3’	This study
		Reverse 5’-ACCACGTTAACTCGTGCAGT-3’	
OG1RF_RS08810		Forward 5’-GCAATGCCCATGTTCTGTTCA-3’	This study
		Reverse 5’-GCGTTCTGGAACAAACCTATCA-3’	

## Results

### *B. subtilis* NTU-18 surfactin production

**Fig. 1:**
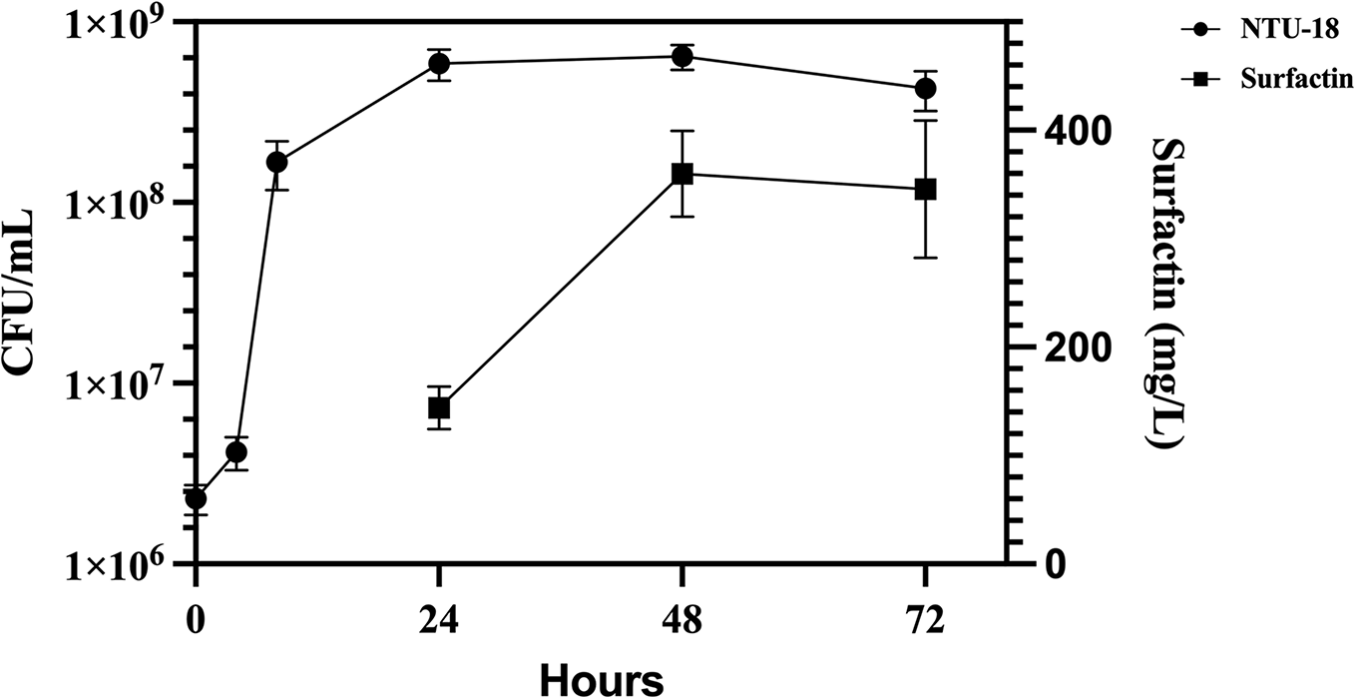
Growth curves of *B. subtilis* natto and surfactin production. *B. subtilis* natto was cultured with 20 g/L yeast extract, 40 g/L glucose, and 2 mM FeSO_4_. The culture was incubated at 37 °C for 72 h with shaking at 120 rpm

As shown in Fig. 1, the population of *B. subtilis* NTU-18 bacteria reached its peak at 6.8 × 10^8^ CFU/mL after 48 h of incubation. Additionally, the production of surfactin increased substantially from 143.8 mg/L at 24 h to 359.8 mg/L at 48 h, where it reached its peak. These data indicate a significant increase in both bacterial growth and surfactin production over the observed period. The marked increase in surfactin concentration between 24 and 48 h suggests that this time frame is critical for optimizing biosurfactant yield in *B. subtilis* cultures.

### LC‒MS analysis of MARs purified from surfactin compounds by NTU-18

**Fig. 2:**
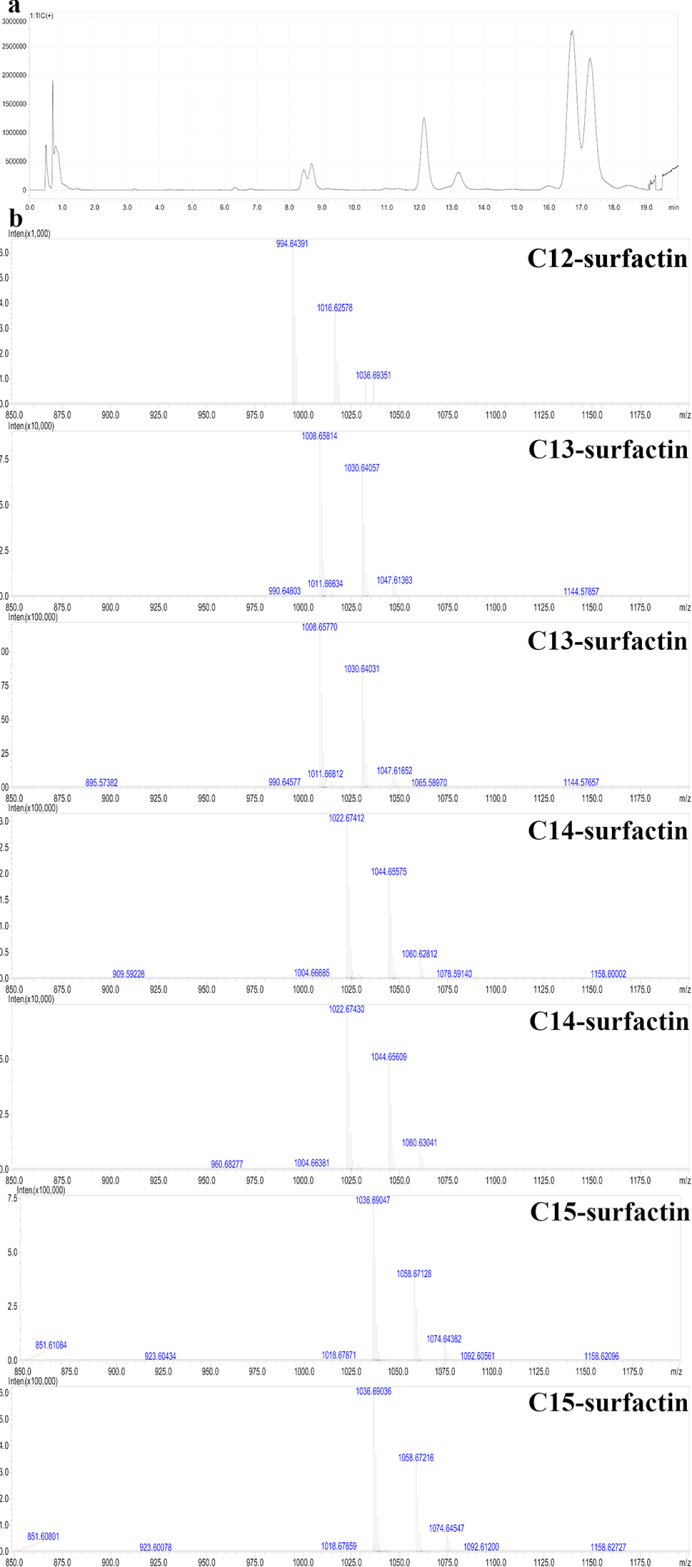
Characterization of surfactin produced by *B. subtilis* natto NTU-18. a. Total ion chromatogram of MARs-purified surfactin. b. Electron ionization mass spectra of MARs-purified surfactin

The total ion chromatogram (TIC) of MARs purified from surfactin is depicted in Fig. 2a. The presence of surfactin produced by *B. subtilis* natto NTU-18 was inferred on the basis of retention times that aligned with those of a surfactin standard. As shown in Supplementary Fig. 1, the TIC and electron ionization mass spectra (EIMS) of the surfactin standard, including seven main isoforms of surfactin, were identified, with retention times (RTs) corresponding to C12-surfactin (6.9 min), C13-surfactin (8.6, 8.9 min), C14-surfactin (12.47 and 13.53 min), and C15-surfactin (17.08 and 17.62 min). In Fig. 2b, [M + H]^+^ signals occurred at m/z 994.64391, 1008.65814, 1008.65770, 1022.67412, 1022.67430, 1036.69047, and 1036.69036. These compounds are homologs of surfactin, with each varying in the length of its β-hydroxy fatty acid chain by a methylene group (CH_2_) of 14 Da.

### Cell viability after treatment with surfactin

**Fig. 3:**
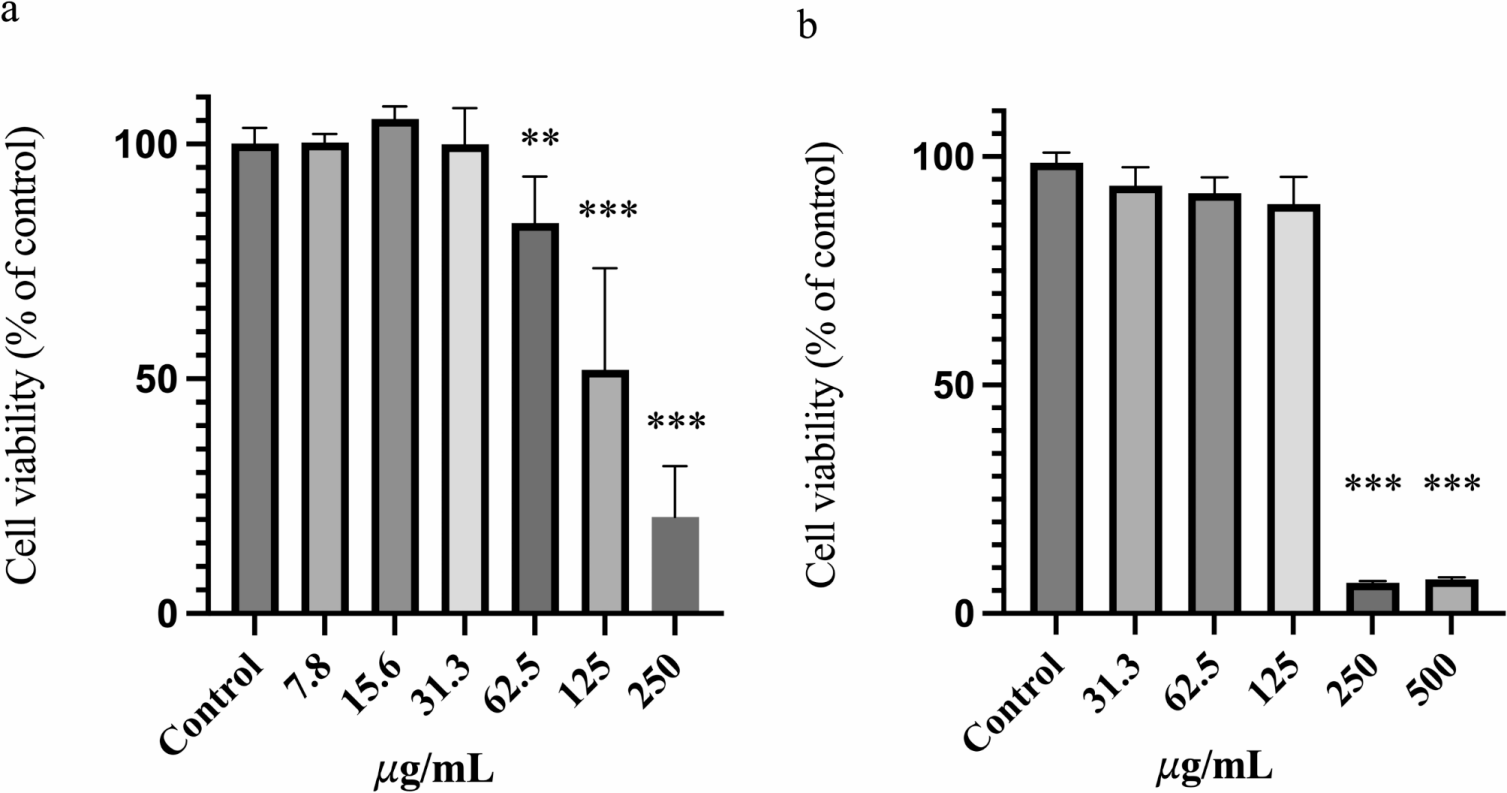
Effects of surfactin on the viability of RAW 264.7 and Caco-2 cells. a. RAW 264.7 and b. Caco-2 cells were treated with various concentrations of MARs-purified surfactin (7.8 to 250 𝜇g/mL and 31.3 to 500 𝜇g/mL) for 48 h. Cell viability was measured via a CCK-8 assay and is presented as a percentage of the control. The data are presented as the means ± SDs (n6). **p<0.01, ***p<0.001 compared with each negative control group (Student's t test)

To identify a concentration of surfactin that is both safe and effective, we evaluated the viability of RAW 264.7 and Caco-2 cells exposed to various concentrations of the extract. Figure 3 shows that no cytotoxic effects were observed in RAW 264.7 cells at surfactin concentrations ranging from 7.8 to 31.3 µg/mL. Consequently, we selected concentrations below 31.3 µg/mL for further experimentation.

### RNA-seq analysis of *E. faecalis* in response to surfactin treatment

**Fig. 4:**
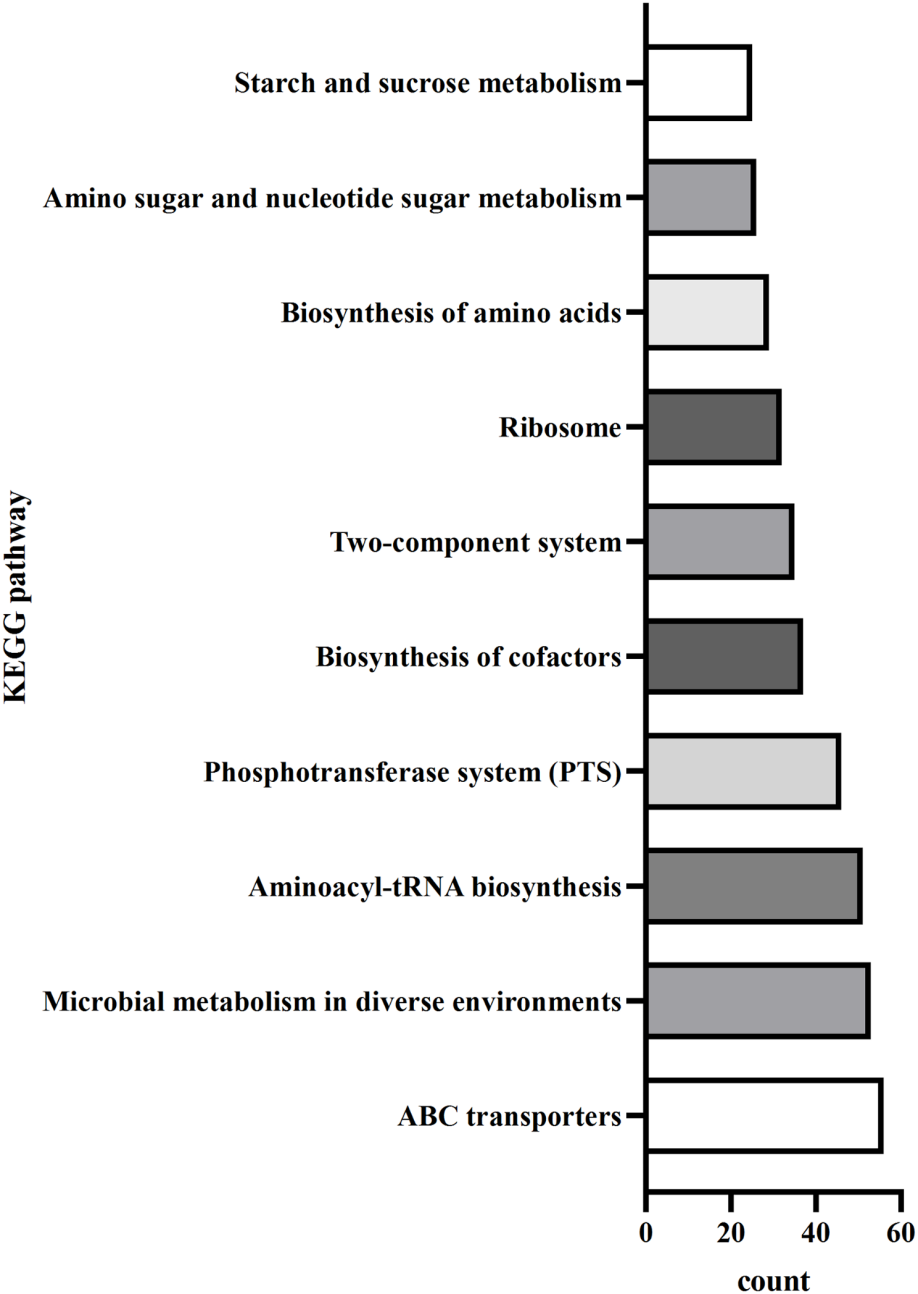
Bar plot of the KEGG pathways enriched in the DEGs affected by surfactin in *E. faecalis*. Each bar represents one of the top 10 pathways. The X-axis shows the number of genes, and the Y-axis shows the pathway classification. Regulated genes were defined as differentially expressed genes with a corrected probability greater than 0.8 and a transcriptional change (either increase or decrease) of more than 1.5-fold in surfactin-treated versus untreated samples

To elucidate the response of *E. faecalis* to surfactin treatment, we identified differentially expressed genes (DEGs) by comparing *E. faecalis* treated with or without surfactin through RNA-seq analysis. Analysis of global gene expression profiles of *E. faecalis* exposed to surfactin, compared with unexposed cells, identified 966 differentially expressed genes (DEGs). Among these genes, 500 showed upregulated expression, whereas 466 showed downregulated expression in the surfactin-treated samples compared to the untreated controls. Kyoto Encyclopedia of Genes and Genomes (KEGG) pathway enrichment analysis was conducted to categorize the DEGs according to their biological functions, as illustrated in Fig. 4. KEGG pathway mapping revealed that the DEGs were associated with amino sugar and exopolysaccharide production (*glnA*, *epaX*, *epaOX*, and OG1RF_RS08810), pilus biosynthesis (*ebpABC*, *ebpR*, and *srtC*), and virulence- and biofilm formation-related genes (*gelE*, *entV* and *bopD*). These findings suggest that surfactin treatment may differentially affect the expression of genes involved in adhesion, aggregation, and biofilm production. To verify the RNA-seq results, we selected 12 DEGs, including pilus-, virulence- and amino sugar- and exopolysaccharide biosynthesis-related genes, for further RT‒qPCR analysis.

### Inhibitory effects of surfactin on virulence factors, pilus formation, and exopolysaccharide synthesis in *E. faecalis*

**Fig. 5:**
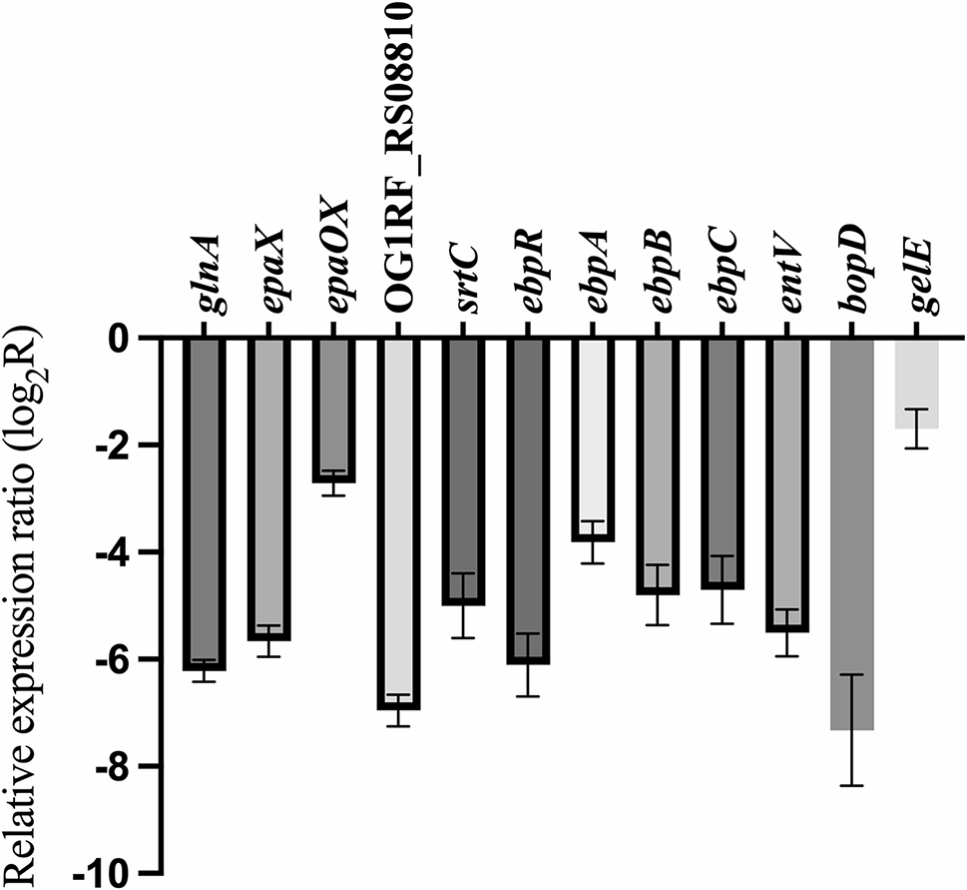
Surfactin inhibits *E. faecalis* polysaccharide-, pilus biosynthesis-, virulence and biofilm-related genes. *E. faecalis *OG1RF was cultured in the presence or absence of surfactin supernatant for 1 h. The data are presented as the means ± SDs (n=3)

To investigate whether surfactin inhibits the expression of virulence factors, pilus synthesis, and exopolysaccharide synthesis, we analyzed the relative expression of genes associated with these processes. As shown in Fig. 5, the expression of amino sugar and exopolysaccharide production genes (*glnA*, *epaX*, *epaOX*, and OG1RF_RS08810), pilus biosynthesis genes (*ebpABC*, *ebpR*, and *srtC*), and virulence and biofilm formation-related genes (*gelE*, *entV*, and *bopD*) was markedly inhibited in the presence of 15.6 µg/mL surfactin. Taken together, these RT‒qPCR results suggest that surfactin may interfere with the synthesis of *E. faecalis* virulence factors pili and exopolysaccharides, thus inhibiting biofilm formation.

### Antibiofilm activity of surfactin against *E. faecalis*

**Fig. 6:**
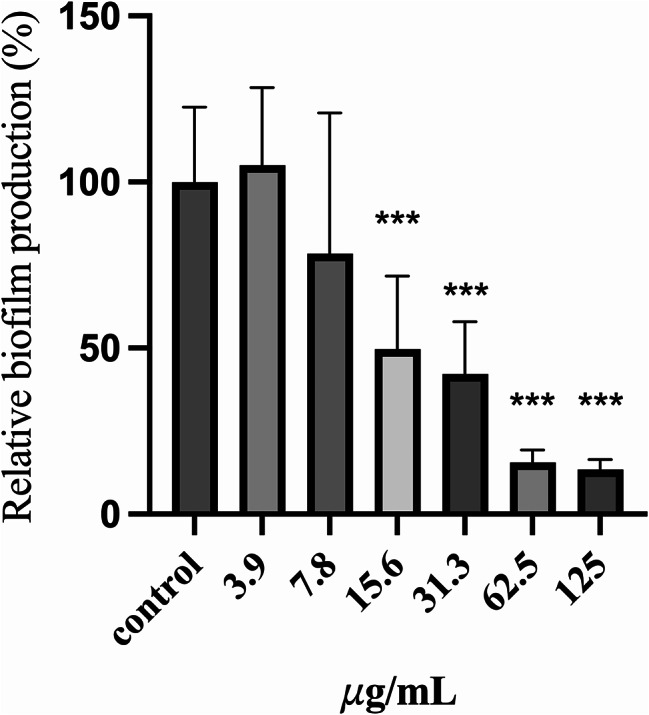
Surfactin interferes with *E. faecalis* biofilm production. Biofilm production in the absence or presence of surfactin for 24 h. Biofilm production is expressed as an index of the biomass stained with safranin (OD450 value) normalized to the cell growth (OD600 value at 24 h). Relative biofilm production (%) was calculated by further normalizing the biofilm index values of the treated group to those of the negative control group without surfactin. The data are presented as the means ± SDs (n$\:\:\ge\:$6). ***p < 0.001 compared with each negative control group (Student’s t test)

To evaluate the impact of surfactin on biofilm formation by *E. faecalis*, we initially determined the MIC of surfactin against *E. faecalis*. We subsequently quantified biofilm production. The MIC of surfactin for *E. faecalis* was determined to be 250 µg/mL. Subsequently, biofilm assays using 96-well polystyrene plates [9] were used to investigate the effect of surfactin on *E. faecalis* biofilm growth after 24 h. Overnight cultures of *E. faecalis* were seeded in TSB medium with or without surfactin and incubated for 24 h. The optical density at 600 nm (OD600) was measured to determine cell growth after the incubation period. Biofilm formation was quantified by measuring the optical density at 450 nm (OD450) of the safranin-stained biomass, which was then normalized to the OD600 values to account for variations in cell growth. As shown in Fig. 6, the biofilm growth of *E. faecalis* at 24 h was significantly inhibited by surfactin treatment. The relative biofilm production rates (%) of *E. faecalis* decreased by 51.2% at a surfactin concentration of 15.6 𝜇g/mL. These results show that surfactin can inhibit *E. faecalis* growth at high concentrations and biofilm production at low concentrations.

### Surfactin influences the adhesion of *E. faecalis* to human intestinal Caco-2 cell monolayers

**Fig. 7:**
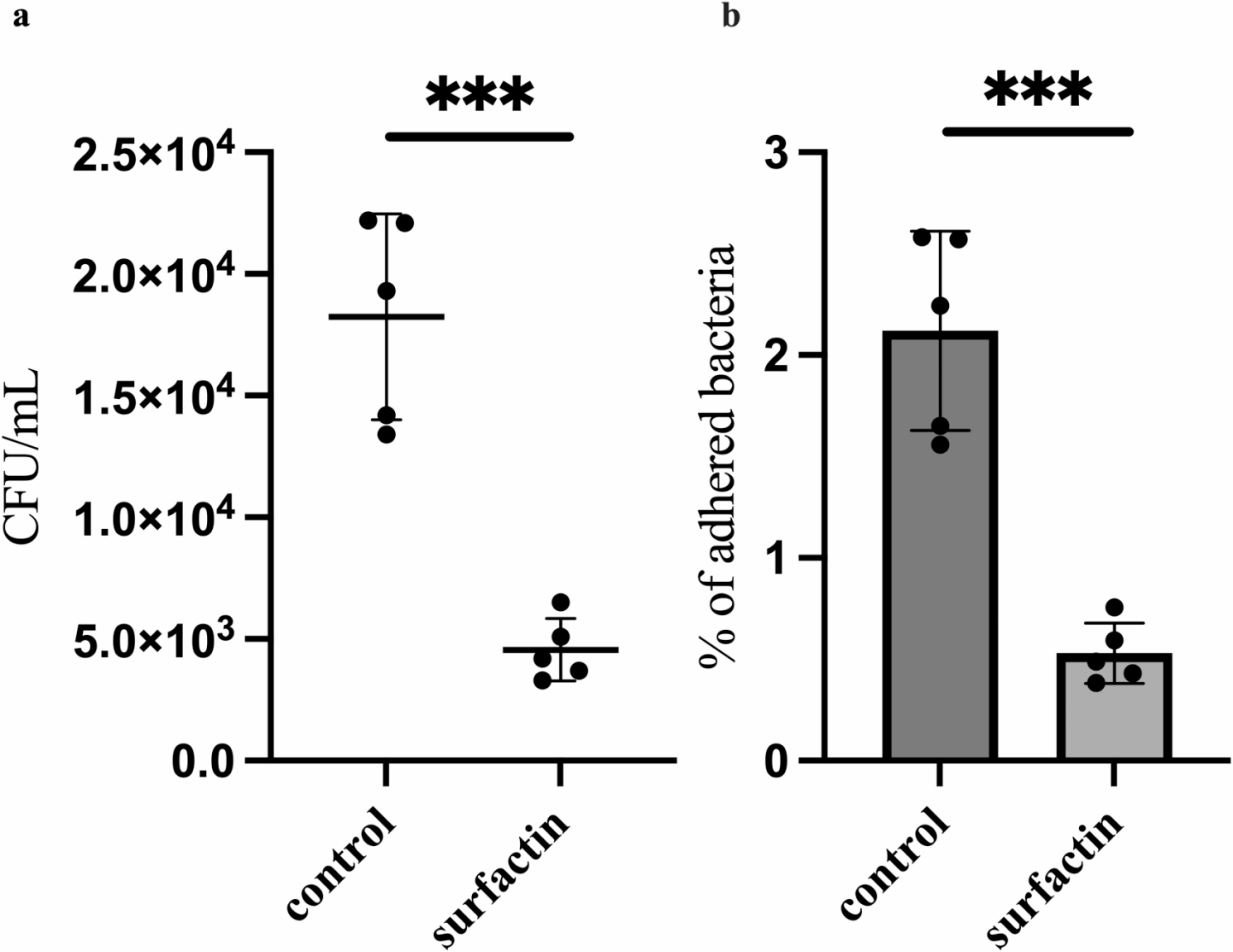
Surfactin affects the adhesion of *E. faecalis* cells to human intestinal Caco-2 cell monolayers. Concentrations a. (CFU/mL) and adhesion rates (%) b. of *E. faecalis* OG1RF adhered to Caco-2 cell monolayers with or without surfactin after 3 h according to an *in vitro* bacterial adhesion assay. In a, each dot in the figures represents a replicate, and the black lines indicate the means ± SDs (n=5). In b, the data are presented as the means ± SDs (n=5). ***p <0.005 compared with each negative control group (Student's t test)

The attachment of bacteria to host tissues and their subsequent colonization are critical, initial steps in biofilm formation [7]. Surfactin has demonstrated efficacy in inhibiting the formation of *E. faecalis* biofilms in vitro. On the basis of these observations, we hypothesized that surfactin hinders the adhesion of *E. faecalis* to Caco-2 human intestinal epithelial cells. We introduced a specified number of *E. faecalis* cells onto Caco-2 cell monolayers in the presence or absence of surfactin. Following the 3 h cocultivation period, the *E. faecalis* cells that had adhered to the Caco-2 monolayers were removed, counted, and cultured on selective medium. As depicted in Fig. 7, the number of *E. faecalis* cells (expressed as colony-forming units (CFUs)/mL) adhering to the cell monolayers significantly decreased following surfactin treatment. The percentage of *E. faecalis* cells adhering to the substrate decreased from 2.12 to 0.53% following treatment with surfactin. This decrease suggests that surfactin effectively interferes with the ability of *E. faecalis* to attach to host tissues, including the human intestinal epithelium. Consequently, these findings underscore the potential utility of surfactin as a therapeutic agent in controlling infections that involve biofilms.

### Surfactin reduces *E. faecalis* aggregates

**Fig. 8:**
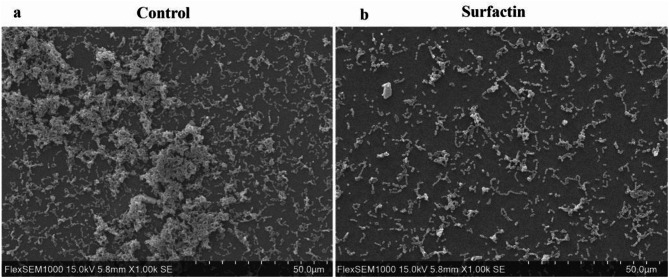
SEM images of *E. faecalis* aggregates. *E. faecalis* OG1RF was grown in the absence (**a**) or presence of surfactin (15.6 µg/mL) (**b**) for 24 h. The aggregates of *E. faecalis* cells were visualized and analyzed via SEM

The morphological characteristics of the bacterial biofilms after 24 h of incubation were examined using SEM. As shown in Fig. 8, the bacterial cells within the control biofilms exhibited dense and thick aggregates, which is indicative of typical biofilm formation. Upon the administration of 15.6 µg/mL surfactin, a notable reduction in the density of these cellular aggregates was observed.

### *E. faecalis* biofilm architecture is impacted by surfactin

**Fig. 9:**
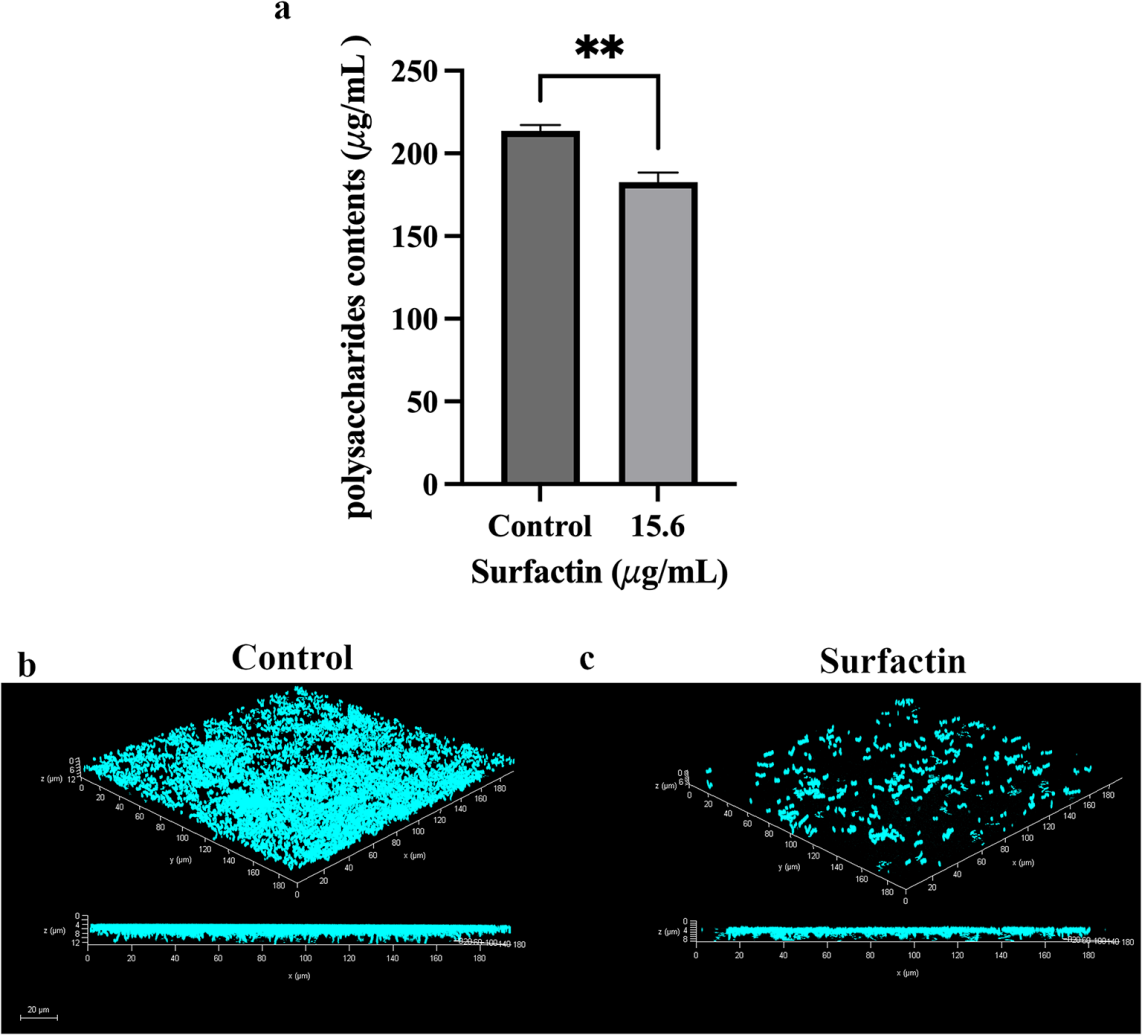
Polysaccharide contents and 3D architecture of *E. faecalis* OG1RF::p23cfp biofilms with or without surfactin. a. Polysaccharide contents in the presence and absence of surfactin (15.6 µg/mL). b. The CFP-labeled strain *E. faecalis* OG1RF::p23cfp was cultured in TSB medium without or with surfactin (15.6 µg/mL) on glass coverslips. The approximate biofilm thicknesses (µm) for all groups were measured and are shown in the figure. The data are presented as the means ± SDs (n ≥3). **p <0.01 compared with each negative control group (Student's t test)

Upon adhering to a solid surface, bacteria are capable of proliferating and secreting an extracellular biofilm matrix composed of extracellular polymeric substances (EPSs), proteins, fatty acids, and nucleic acids [7, 39]. EPSs are critical for forming the three-dimensional structures of biofilms, constituting more than 90% of their dry masses in many cases. Additionally, polysaccharides represent the primary constituents of EPSs [40]. Therefore, the control of biofilm development is crucially dependent on the inhibition or reduction in EPS production. The EPS contents in the control and surfactin-treated *E. faecalis* biofilms were quantified via an assay. As depicted in Fig. 9a, the administration of 15.6 µg/mL surfactin resulted in a significant reduction in the EPS content by 14.5% in *E. faecalis* biofilms. Consequently, treatment with 15.6 µg/mL surfactin effectively inhibited the production of EPS within biofilms by *E. faecalis*.

Alterations in the 3D architecture of *E. faecalis* OG1RF::p23cfp biofilms, corresponding to changes in the extrapolymeric matrix content in the presence of surfactin, were visualized via confocal laser scanning microscopy (CLSM). The biofilms that developed on glass coverslips positioned at the bottoms of the tissue culture dishes were examined in the presence and in the absence of surfactin. The results are shown in Fig. 9b and c. After 24 h of cultivation, the biofilms formed by the control group of *E. faecalis* were dense and well structured. In contrast, biofilms from the surfactin-treated groups were less dense and displayed a more disorganized structure. Additionally, surfactin-treated *E. faecalis* biofilms were thinner, measuring approximately 4 μm in thickness, than the 8 μm thickness observed in the control biofilms. These findings collectively suggest that surfactin not only inhibits the production of the extrapolymeric matrix within biofilms by bacterial cells but also leads to the formation of looser and thinner biofilms.

## Discussion

Pathogenic biofilms have gained attention as critical targets for the treatment of bacterial infections because of their involvement in a wide range of bacterial disease processes. Once bacteria become embedded in biofilms, they are inherently less susceptible to antimicrobial agents than motile cells in a planktonic state are [41], and their antibiotic resistance increases [42]. A previous study demonstrated the therapeutic capabilities of surfactin in various animal models. One study demonstrated that surfactin induces regulatory T-cells (Tregs) to inhibit chronic inflammation and attenuate atherosclerosis in mice [43]. Furthermore, surfactin has been shown to mitigate hyperglycemia in mice treated by a combination of a high-fat diet and streptozotocin to induce type 2 diabetes [44]. Surfactin also improved intestinal dysbiosis, alleviated inflammation in the colon and brain, and mitigated behavioral disorders in mice with DSS-induced colitis [45]. These studies demonstrate the therapeutic potential of surfactin for various diseases.

In this study, our research demonstrated that surfactin affects expression of biofilm biosynthesis-related genes in *E. faecalis* and significantly inhibits adherence to Caco-2 cell monolayers, as well as the capacity for aggregation and biofilm production, thus impairing *E. faecalis* biofilm formation. Via RNA-seq and RT‒qPCR analyses, we found that surfactin treatment inhibited the expression of genes involved in polysaccharide production, including *glnA*, *epaX*, *epaOX*, and OG1RF_RS08810. The *glnA* gene encodes a type I glutamate-ammonia ligase, which catalyzes the conversion of glutamate and ammonia into glutamine [46]. The *epaX* gene encodes a glycosyltransferase within the enterococcal polysaccharide antigen (EPA) locus, altering the composition of exopolysaccharides to increase bile salt resistance and cell wall integrity [47]. The *glnA* and *epaX* genes are involved in the synthesis of β-1,6-linked poly-N-acetylglucosamine (polyGlcNAc), which endows *E. faecalis* with the ability to penetrate surfaces [46]. Expression of the *epaOX* and OG1RF_RS08810 genes results in pronounced impacts on the structure and productivity of exopolysaccharides [39, 48]. Following treatment with surfactin, we observed a significant reduction in extracellular polysaccharide levels. In addition to exopolysaccharide-related genes, expression of virulence-related genes, including biofilm on plastic surfaces (*bopD*) and gelatinase (*gelE*), was inhibited in *E. faecalis* treated with surfactin. Gelatinase, an extracellular metalloprotease that can hydrolyze gelatin, collagen, and hemoglobin, is also known to play a role in bacterial adherence, biofilm formation, and bacterial translocation [49–51]. Surfactin inhibits the *ebpABC* component of the *ebp* operon, which is crucial for encoding the pili associated with endocarditis and biofilm formation. This inhibition by surfactin impacts the aggregation and adherence capabilities of *E. faecalis* [52, 53]. Flores-Mireles et al. [54] revealed that upon catheter insertion into mouse bladders, fibrinogen released from host cells serves as a substrate for *E. faecalis*, which utilizes the adhesin EbpA to bind to host fibrinogen and subsequently forms biofilms on the catheter by consuming fibrinogen. Collectively, these studies provide evidence of the importance of exopolysaccharides, virulence factors, and bacterial pilus biosynthesis in biofilm formation. These findings support the idea that surfactin may have antibiofilm activity against *E. faecalis*. In support of this concept, surfactin has been demonstrated to inhibit biofilm formation across various microbial pathogens [55–57]. In 2019, Liu et al. [55] reported that surfactin influences the quorum sensing (QS) mechanism in *Staphylococcus aureus* by modulating the activity of autoinducer 2 (AI-2), which consequently impacts biofilm formation. In 2020, Janek et al. [56] reported that surfactin and metal (II)-surfactin complexes may serve as potential antibiofilm agents against hypha-related *Candida albicans* infections in clinical settings. In 2024, Joeng et al. [58] conducted a comprehensive study and reported that surfactin exhibited antibacterial and antibiofilm activity against methicillin-resistant *S. aureus* (MRSA). There are some similarities in the inhibition of related gene expression; however, it appears that extracellular polysaccharide biosynthesis-related genes were not specifically examined in their study. Additionally, their surfactin showed m/z values of 1022.676 and 1036.693, while our strain can produce surfactin with shorter carbon chains. Conducting antibiofilm experiments with surfactin of different carbon chain lengths may help to elucidate their functional differences.

Surfactin, a biosurfactant produced by *Bacillus* species, holds potential applications in bioremediation, food safety, and agriculture due to its amphiphilic properties [59], which enable effective emulsification and solubilization of hydrophobic pollutants [60]. Its application in the food and agriculture industries shows promise due to its emulsion-forming, antiadhesive, and functional ingredient properties [61]. Surfactin enhances the bioremediation process by increasing the solubility and bioavailability of hydrophobic pollutants in soil [62]. Additionally, surfactin exhibits exceptional antagonistic efficacy against *Fusarium moniliforme*-caused bakanae disease [63]. Jeong et al. [58] also pointed out that surfactin does not pose a risk to the environment. Therefore, surfactin demonstrates significant potential in practical applications. However, further studies using surfactin produced by *B. subtilis natto* NTU-18 are necessary, particularly in the agricultural sector, to expand its broader applicability.

Another major lipopeptide produced by the *Bacillus* genus is fengycin, which comprises cyclic decapeptides attached to a β-hydroxy fatty acid chain via an internal ester bond. The fengycin family includes two main variants: fengycin A and fengycin B [64]. Compared with surfactin, fengycin exhibits effective antifungal activity, particularly against filamentous fungi, while showing limited efficacy against yeast and bacteria [65]. Fengycin is currently widely utilized in the management of fungal infections. Its mode of action involves inhibiting the growth and development of pathogenic fungi, thereby providing protection to plants against fungal diseases. Fengycin has been identified as the primary antifungal agent produced by *B. subtilis* NCD-2, demonstrating activity against *Rhizoctonia solani*, the pathogen responsible for damping-off in cotton [66]. Fengycins produced by *B. velezensis* FJAT have been identified as the predominant lipopeptides responsible for antibacterial activity against *Ralstonia solanacearum*, the causative agent of bacterial wilt in tomatoes [67]. Fengycin exerts its antifungal effects by compromising the integrity of fungal cell membranes, resulting in cell lysis and eventual death [68]. Overall, lipopeptides have significant potential for development in biomedical and biocontrol applications. However, further research is needed to determine whether surfactin or fengycin may contribute to the development of microbial resistance.

Our findings indicate that surfactin significantly inhibits the biofilm formation of *E. faecalis*. Surfactin may interfere with exopolysaccharide production, virulence factor expression, and pilus biosynthesis, thereby impairing the biofilm formation ability of *E. faecalis*. On the basis of these results, surfactin has potential for use in managing infections associated with *E. faecalis* biofilms.

## Electronic supplementary material

Below is the link to the electronic supplementary material.

Supplementary Material 1

## Data Availability

The RNA-seq datasets generated and/or analysed during the current study are available in the NCBI’s Gene Expression Omnibus repository, and are accessible at GEO series accession number GSE280692 (https://www.ncbi.nlm.nih.gov/geo/query/acc.cgi?%26acc=GSE280692).
