# Supplementary material for: Surfactin inhibits enterococcal biofilm formation via interference with pilus and exopolysaccharide biosynthesis
Source: BMC Microbiol. 2025 Feb 24;25:85. doi: 10.1186/s12866-025-03786-y (PMC11852883; doi:10.1186/s12866-025-03786-y)
Supplement: Supplementary file 1 — Supplementary Material 1 [file 12866_2025_3786_MOESM1_ESM.docx]

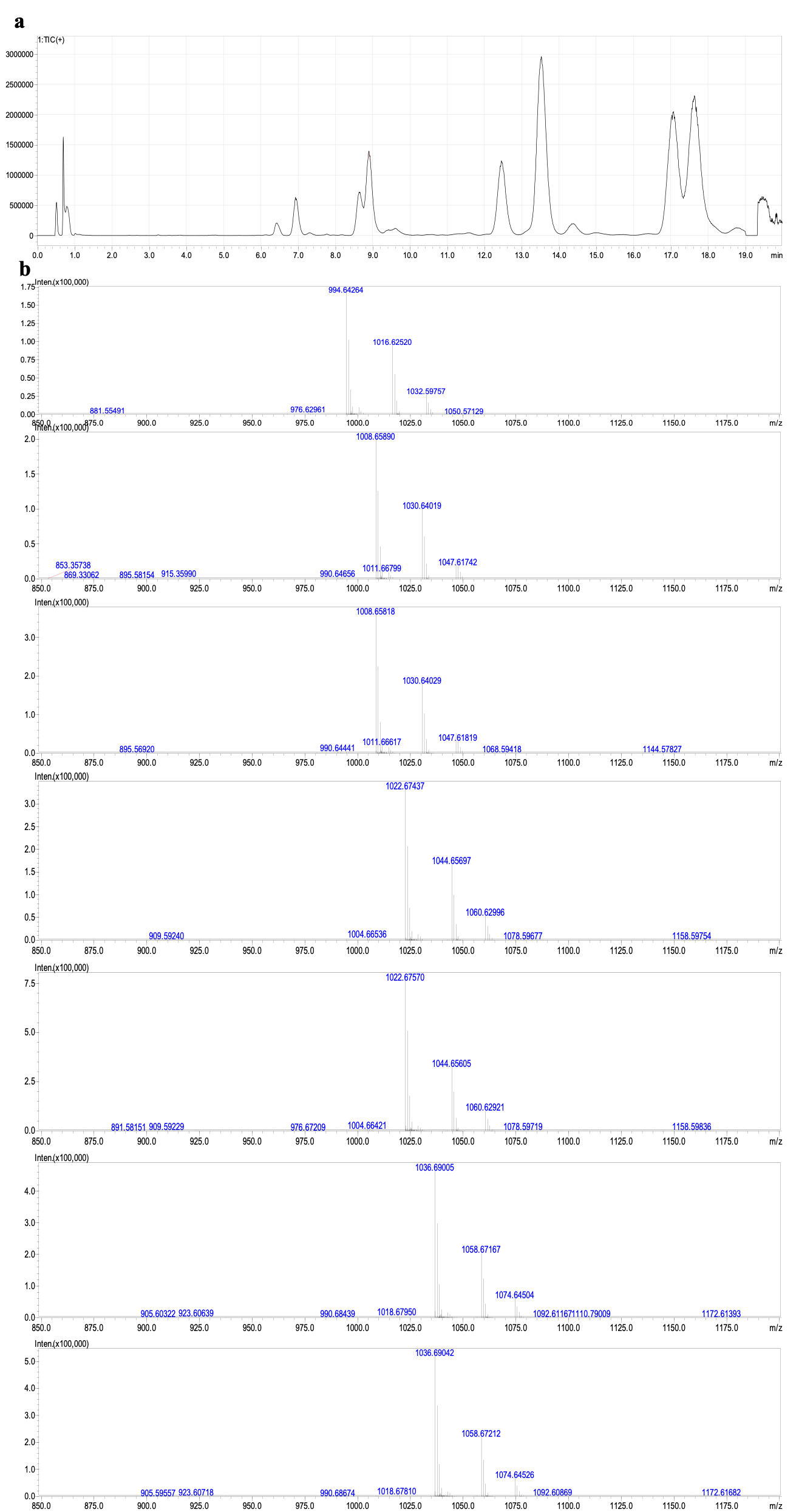


**a**

Supplementary Fig. 1 Characterization of surfactin standard

a. Total ion chromatogram of surfactin standard. b. Electron ionization mass spectra of surfactin standard.
